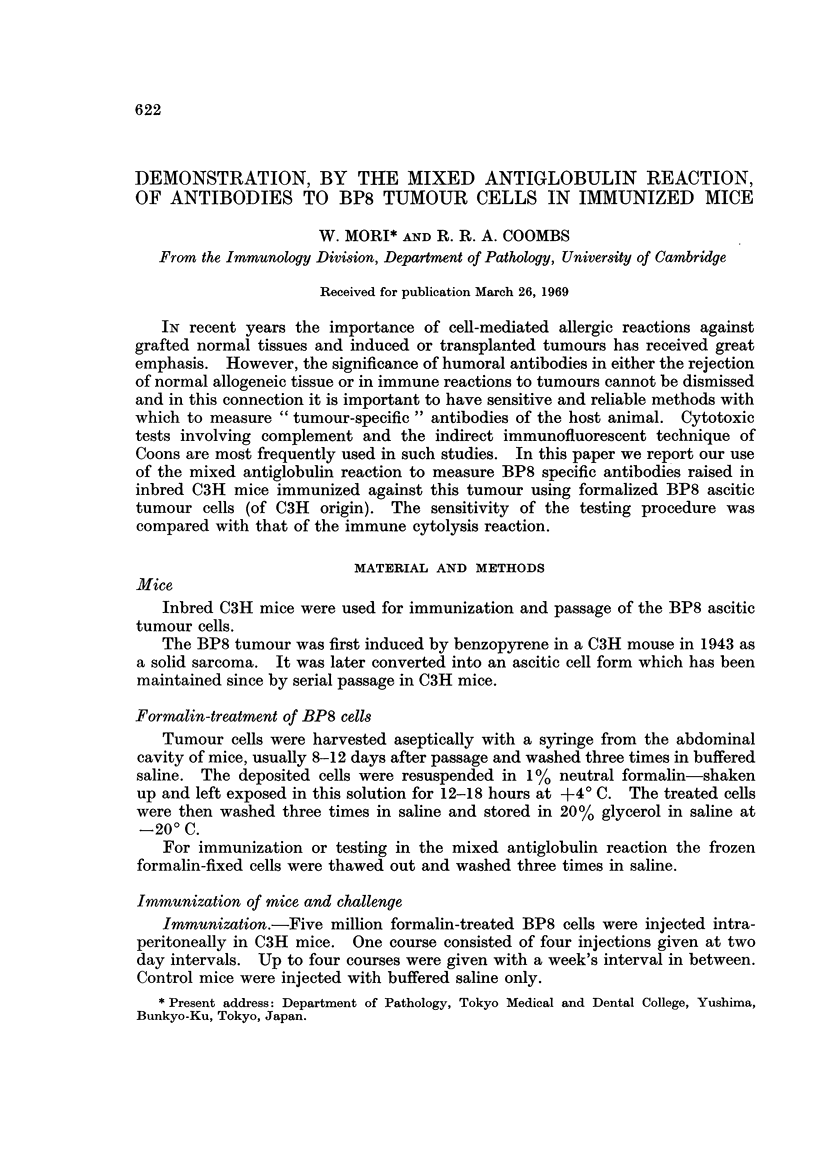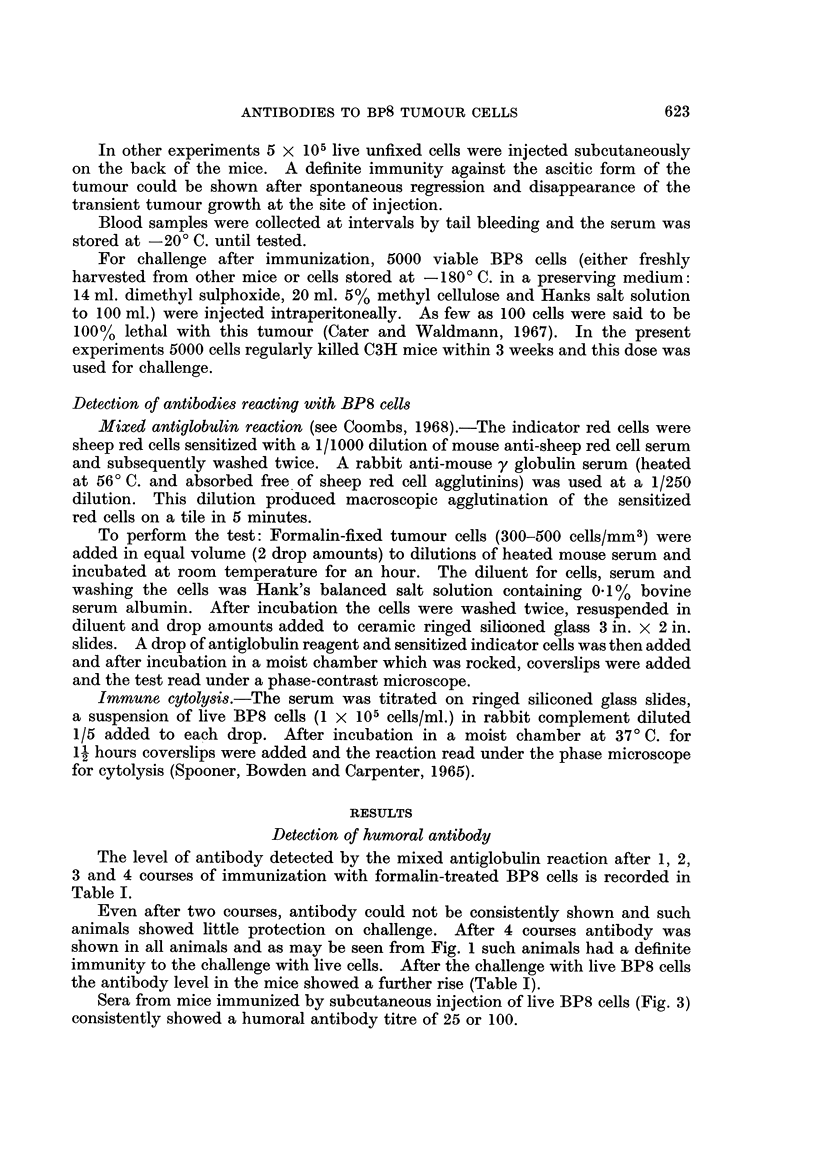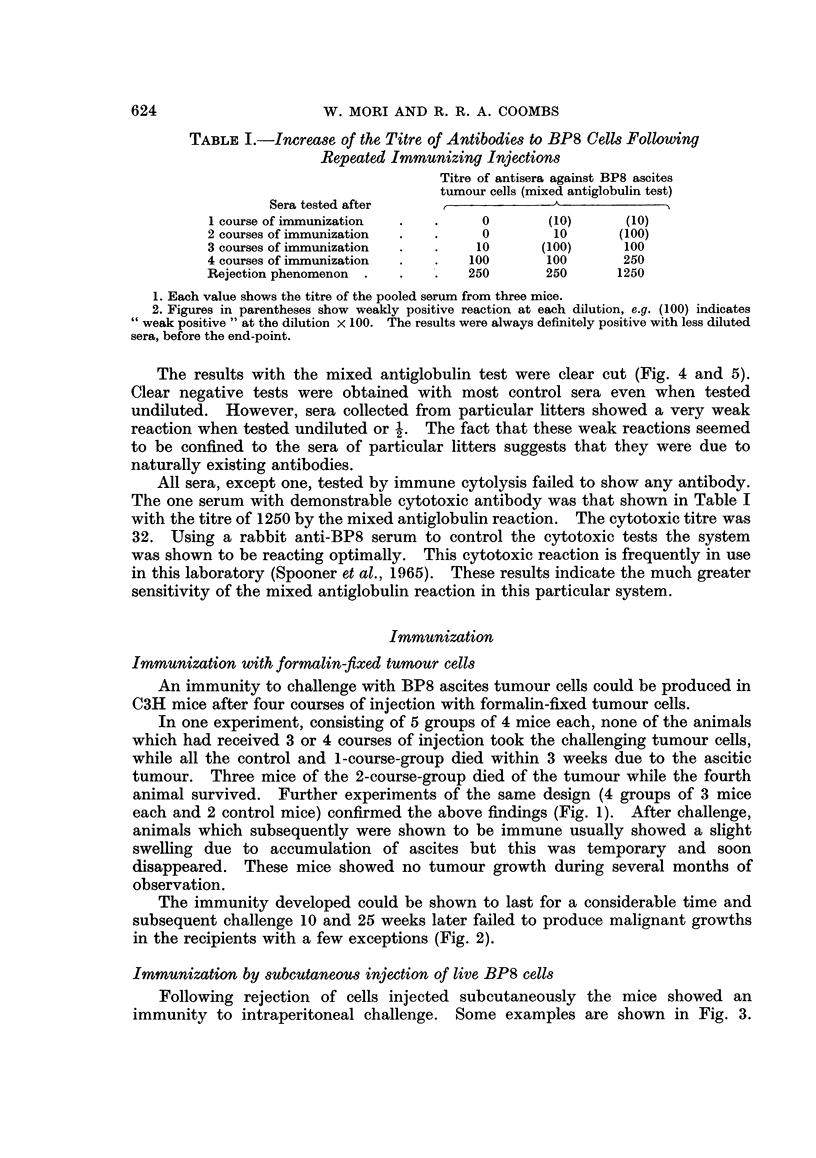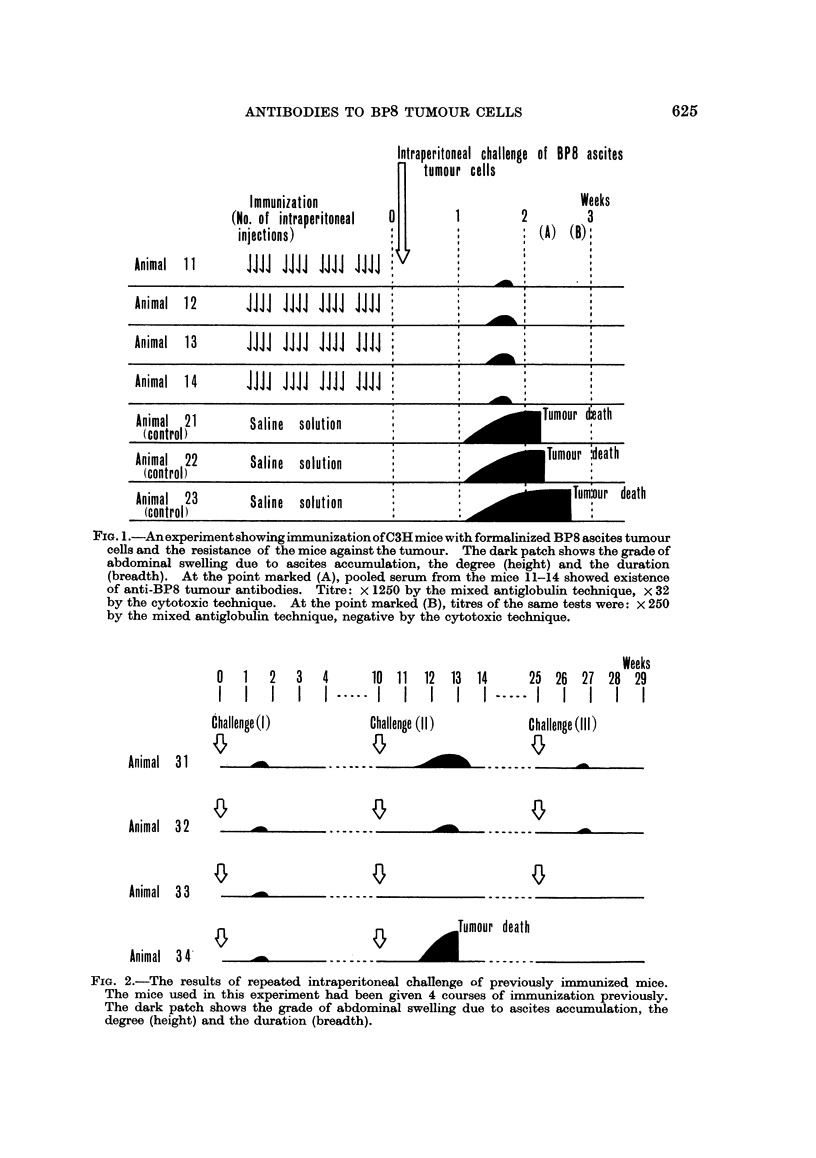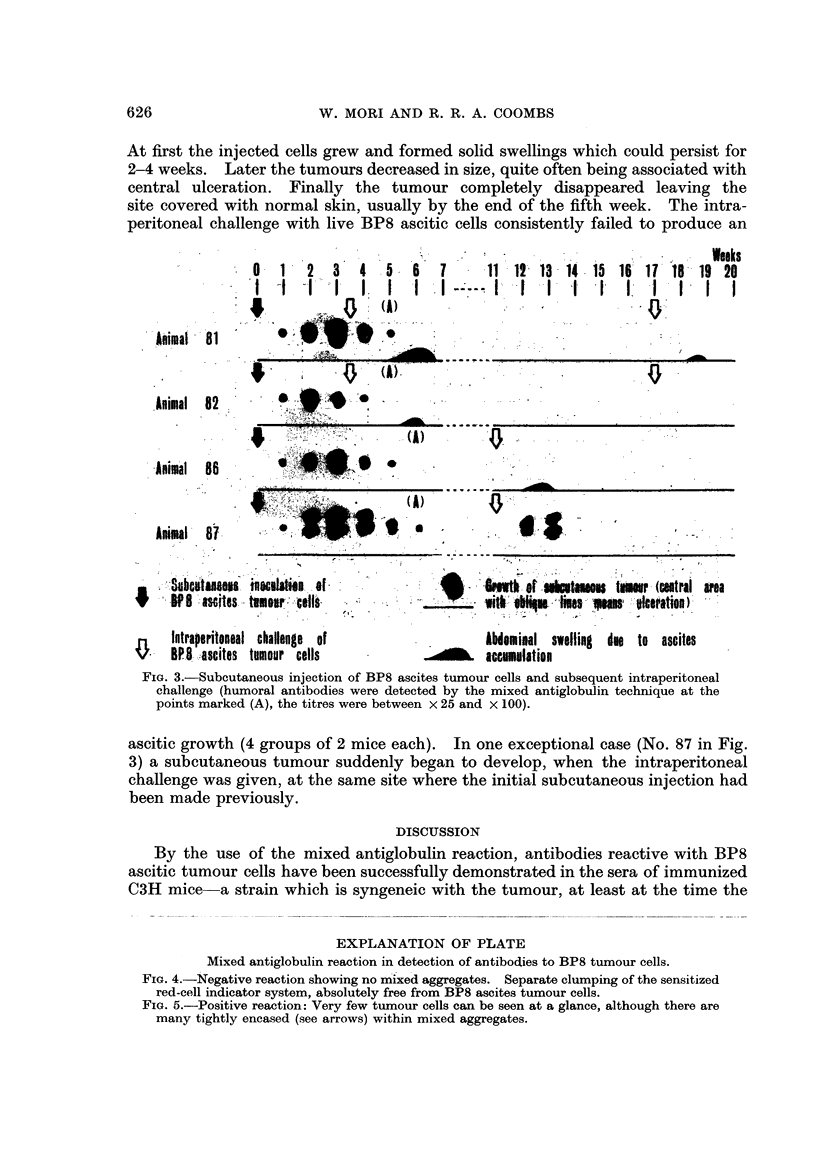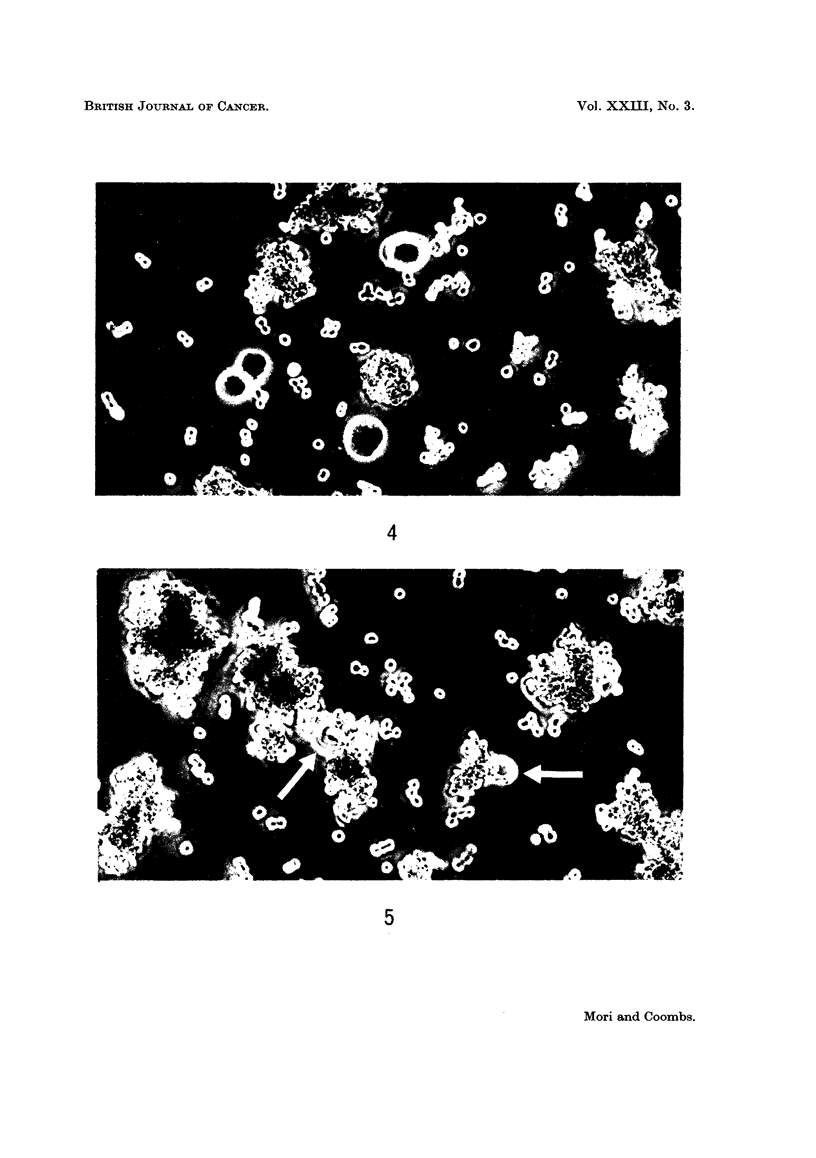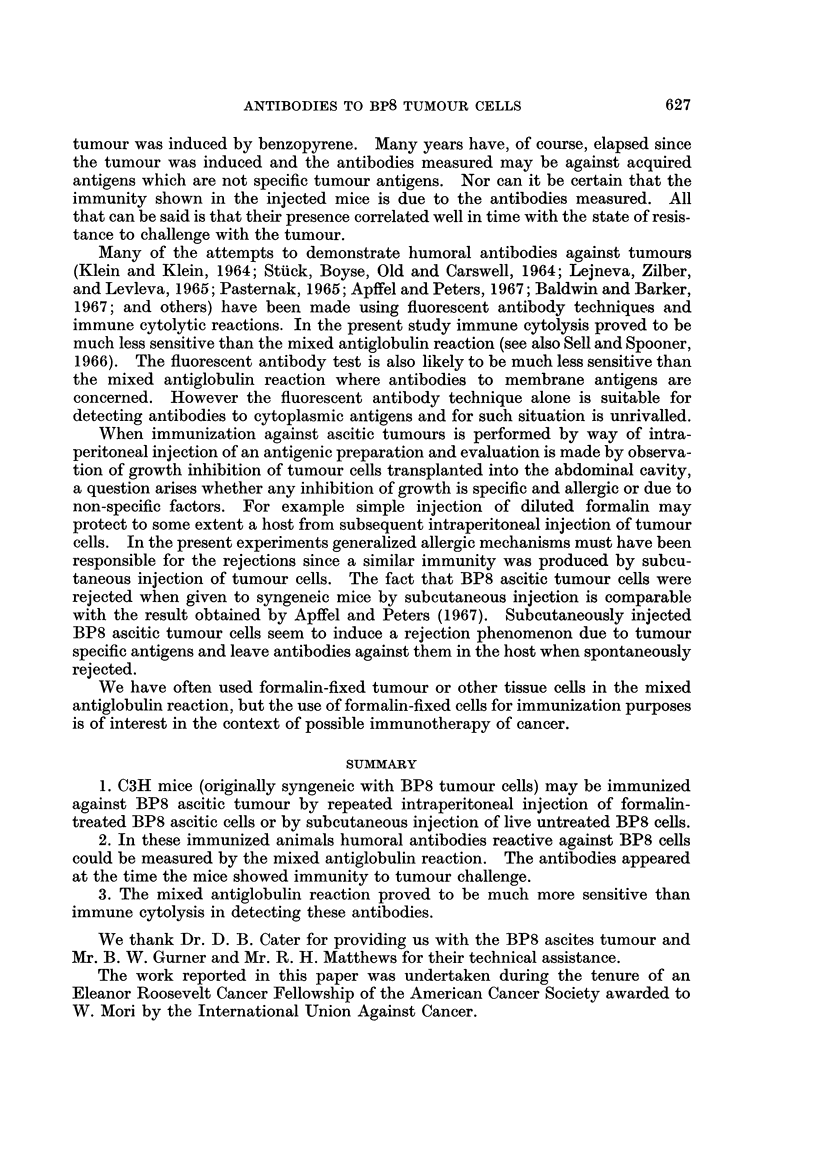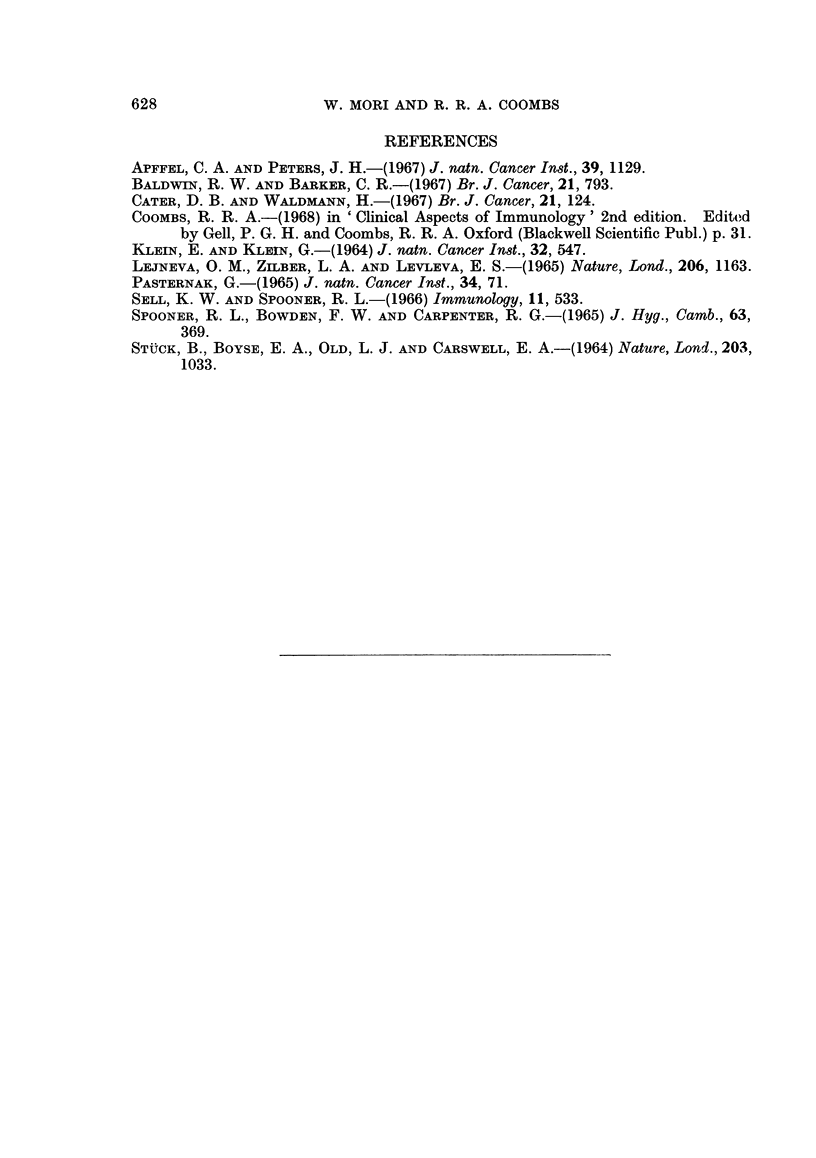# Demonstration by the mixed antiglobulin reaction, of antibodies to BP8 tumour cells in immunized mice.

**DOI:** 10.1038/bjc.1969.76

**Published:** 1969-09

**Authors:** W. Mori, R. R. Coombs

## Abstract

**Images:**


### DEMONSTRATION, BY THE MIXED ANTIGLOBULIN REACTION, OF ANTIBODIES TO BP8 TUMOUR CELLS IN IMMUNIZED MICE

W. MORI* AND R. R. A. COOMBS

*From the Immunology Division, Department of Pathology, University of Cambridge*

Received for publication March 26, 1969

IN recent years the importance of cell-mediated allergic reactions against grafted normal tissues and induced or transplanted tumours has received great emphasis. However, the significance of humoral antibodies in either the rejection of normal allogeneic tissue or in immune reactions to tumours cannot be dismissed and in this connection it is important to have sensitive and reliable methods with which to measure "tumour-specific" antibodies of the host animal. Cytotoxic tests involving complement and the indirect immunofluorescent technique of Coons are most frequently used in such studies. In this paper we report our use of the mixed antiglobulin reaction to measure BP8 specific antibodies raised in inbred C3H mice immunized against this tumour using formalized BP8 ascitic tumour cells (of C3H origin). The sensitivity of the testing procedure was compared with that of the immune cytolysis reaction.

### MATERIAL AND METHODS

### *Mice*

Inbred C3H mice were used for immunization and passage of the BP8 ascitic tumour cells.

The BP8 tumour was first induced by benzopyrene in a C3H mouse in 1943 as a solid sarcoma. It was later converted into an ascitic cell form which has been maintained since by serial passage in C3H mice.

### *Formalin-treatment of BP8 cells*

Tumour cells were harvested aseptically with a syringe from the abdominal cavity of mice, usually 8–12 days after passage and washed three times in buffered saline. The deposited cells were resuspended in 1% neutral formalin—shaken up and left exposed in this solution for 12–18 hours at +4° C. The treated cells were then washed three times in saline and stored in 20% glycerol in saline at −20° C.

For immunization or testing in the mixed antiglobulin reaction the frozen formalin-fixed cells were thawed out and washed three times in saline.

### *Immunization of mice and challenge*

*Immunization.*—Five million formalin-treated BP8 cells were injected intraperitoneally in C3H mice. One course consisted of four injections given at two day intervals. Up to four courses were given with a week's interval in between. Control mice were injected with buffered saline only.

* Present address: Department of Pathology, Tokyo Medical and Dental College, Yushima, Bunkyo-Ku, Tokyo, Japan.

In other experiments $5 \times 10^5$ live unfixed cells were injected subcutaneously on the back of the mice. A definite immunity against the ascitic form of the tumour could be shown after spontaneous regression and disappearance of the transient tumour growth at the site of injection.

Blood samples were collected at intervals by tail bleeding and the serum was stored at —20° C. until tested.

For challenge after immunization, 5000 viable BP8 cells (either freshly harvested from other mice or cells stored at —180° C. in a preserving medium: 14 ml. dimethyl sulphoxide, 20 ml. 5% methyl cellulose and Hanks salt solution to 100 ml.) were injected intraperitoneally. As few as 100 cells were said to be 100% lethal with this tumour (Cater and Waldmann, 1967). In the present experiments 5000 cells regularly killed C3H mice within 3 weeks and this dose was used for challenge.

*Detection of antibodies reacting with BP8 cells*

*Mixed antiglobulin reaction* (see Coombs, 1968).—The indicator red cells were sheep red cells sensitized with a 1/1000 dilution of mouse anti-sheep red cell serum and subsequently washed twice. A rabbit anti-mouse $\gamma$ globulin serum (heated at 56° C. and absorbed free of sheep red cell agglutinins) was used at a 1/250 dilution. This dilution produced macroscopic agglutination of the sensitized red cells on a tile in 5 minutes.

To perform the test: Formalin-fixed tumour cells (300–500 cells/mm$^3$) were added in equal volume (2 drop amounts) to dilutions of heated mouse serum and incubated at room temperature for an hour. The diluent for cells, serum and washing the cells was Hank's balanced salt solution containing 0·1% bovine serum albumin. After incubation the cells were washed twice, resuspended in diluent and drop amounts added to ceramic ringed siliconed glass 3 in. × 2 in. slides. A drop of antiglobulin reagent and sensitized indicator cells was then added and after incubation in a moist chamber which was rocked, coverslips were added and the test read under a phase-contrast microscope.

*Immune cytolysis.*—The serum was titrated on ringed siliconed glass slides, a suspension of live BP8 cells ($1 \times 10^5$ cells/ml.) in rabbit complement diluted 1/5 added to each drop. After incubation in a moist chamber at 37° C. for $1\frac{1}{2}$ hours coverslips were added and the reaction read under the phase microscope for cytolysis (Spooner, Bowden and Carpenter, 1965).

### RESULTS

### *Detection of humoral antibody*

The level of antibody detected by the mixed antiglobulin reaction after 1, 2, 3 and 4 courses of immunization with formalin-treated BP8 cells is recorded in Table I.

Even after two courses, antibody could not be consistently shown and such animals showed little protection on challenge. After 4 courses antibody was shown in all animals and as may be seen from Fig. 1 such animals had a definite immunity to the challenge with live cells. After the challenge with live BP8 cells the antibody level in the mice showed a further rise (Table I).

Sera from mice immunized by subcutaneous injection of live BP8 cells (Fig. 3) consistently showed a humoral antibody titre of 25 or 100.

TABLE I.—*Increase of the Titre of Antibodies to BP8 Cells Following Repeated Immunizing Injections*

| Sera tested after | Titre of antisera against BP8 ascites tumour cells (mixed antiglobulin test) | | |
|---|---|---|---|
| 1 course of immunization | 0 | (10) | (10) |
| 2 courses of immunization | 0 | 10 | (100) |
| 3 courses of immunization | 10 | (100) | 100 |
| 4 courses of immunization | 100 | 100 | 250 |
| Rejection phenomenon | 250 | 250 | 1250 |

1. Each value shows the titre of the pooled serum from three mice.
2. Figures in parentheses show weakly positive reaction at each dilution, *e.g.* (100) indicates " weak positive " at the dilution × 100. The results were always definitely positive with less diluted sera, before the end-point.

The results with the mixed antiglobulin test were clear cut (Fig. 4 and 5). Clear negative tests were obtained with most control sera even when tested undiluted. However, sera collected from particular litters showed a very weak reaction when tested undiluted or $\frac{1}{2}$. The fact that these weak reactions seemed to be confined to the sera of particular litters suggests that they were due to naturally existing antibodies.

All sera, except one, tested by immune cytolysis failed to show any antibody. The one serum with demonstrable cytotoxic antibody was that shown in Table I with the titre of 1250 by the mixed antiglobulin reaction. The cytotoxic titre was 32. Using a rabbit anti-BP8 serum to control the cytotoxic tests the system was shown to be reacting optimally. This cytotoxic reaction is frequently in use in this laboratory (Spooner *et al.*, 1965). These results indicate the much greater sensitivity of the mixed antiglobulin reaction in this particular system.

### *Immunization*

### *Immunization with formalin-fixed tumour cells*

An immunity to challenge with BP8 ascites tumour cells could be produced in C3H mice after four courses of injection with formalin-fixed tumour cells.

In one experiment, consisting of 5 groups of 4 mice each, none of the animals which had received 3 or 4 courses of injection took the challenging tumour cells, while all the control and 1-course-group died within 3 weeks due to the ascitic tumour. Three mice of the 2-course-group died of the tumour while the fourth animal survived. Further experiments of the same design (4 groups of 3 mice each and 2 control mice) confirmed the above findings (Fig. 1). After challenge, animals which subsequently were shown to be immune usually showed a slight swelling due to accumulation of ascites but this was temporary and soon disappeared. These mice showed no tumour growth during several months of observation.

The immunity developed could be shown to last for a considerable time and subsequent challenge 10 and 25 weeks later failed to produce malignant growths in the recipients with a few exceptions (Fig. 2).

### *Immunization by subcutaneous injection of live BP8 cells*

Following rejection of cells injected subcutaneously the mice showed an immunity to intraperitoneal challenge. Some examples are shown in Fig. 3.

FIG. 1.—An experiment showing immunization of C3H mice with formalinized BP8 ascites tumour cells and the resistance of the mice against the tumour. The dark patch shows the grade of abdominal swelling due to ascites accumulation, the degree (height) and the duration (breadth). At the point marked (A), pooled serum from the mice 11–14 showed existence of anti-BP8 tumour antibodies. Titre: ×1250 by the mixed antiglobulin technique, ×32 by the cytotoxic technique. At the point marked (B), titres of the same tests were: ×250 by the mixed antiglobulin technique, negative by the cytotoxic technique.

FIG. 2.—The results of repeated intraperitoneal challenge of previously immunized mice. The mice used in this experiment had been given 4 courses of immunization previously. The dark patch shows the grade of abdominal swelling due to ascites accumulation, the degree (height) and the duration (breadth).

At first the injected cells grew and formed solid swellings which could persist for 2–4 weeks. Later the tumours decreased in size, quite often being associated with central ulceration. Finally the tumour completely disappeared leaving the site covered with normal skin, usually by the end of the fifth week. The intraperitoneal challenge with live BP8 ascitic cells consistently failed to produce an

FIG. 3.—Subcutaneous injection of BP8 ascites tumour cells and subsequent intraperitoneal challenge (humoral antibodies were detected by the mixed antiglobulin technique at the points marked (A), the titres were between $\times 25$ and $\times 100$).

ascitic growth (4 groups of 2 mice each). In one exceptional case (No. 87 in Fig. 3) a subcutaneous tumour suddenly began to develop, when the intraperitoneal challenge was given, at the same site where the initial subcutaneous injection had been made previously.

### DISCUSSION

By the use of the mixed antiglobulin reaction, antibodies reactive with BP8 ascitic tumour cells have been successfully demonstrated in the sera of immunized C3H mice—a strain which is syngeneic with the tumour, at least at the time the

### EXPLANATION OF PLATE

Mixed antiglobulin reaction in detection of antibodies to BP8 tumour cells.

FIG. 4.—Negative reaction showing no mixed aggregates. Separate clumping of the sensitized red-cell indicator system, absolutely free from BP8 ascites tumour cells.

FIG. 5.—Positive reaction: Very few tumour cells can be seen at a glance, although there are many tightly encased (see arrows) within mixed aggregates.

4

5

tumour was induced by benzopyrene. Many years have, of course, elapsed since the tumour was induced and the antibodies measured may be against acquired antigens which are not specific tumour antigens. Nor can it be certain that the immunity shown in the injected mice is due to the antibodies measured. All that can be said is that their presence correlated well in time with the state of resistance to challenge with the tumour.

Many of the attempts to demonstrate humoral antibodies against tumours (Klein and Klein, 1964; Stück, Boyse, Old and Carswell, 1964; Lejneva, Zilber, and Levleva, 1965; Pasternak, 1965; Apffel and Peters, 1967; Baldwin and Barker, 1967; and others) have been made using fluorescent antibody techniques and immune cytolytic reactions. In the present study immune cytolysis proved to be much less sensitive than the mixed antiglobulin reaction (see also Sell and Spooner, 1966). The fluorescent antibody test is also likely to be much less sensitive than the mixed antiglobulin reaction where antibodies to membrane antigens are concerned. However the fluorescent antibody technique alone is suitable for detecting antibodies to cytoplasmic antigens and for such situation is unrivalled.

When immunization against ascitic tumours is performed by way of intraperitoneal injection of an antigenic preparation and evaluation is made by observation of growth inhibition of tumour cells transplanted into the abdominal cavity, a question arises whether any inhibition of growth is specific and allergic or due to non-specific factors. For example simple injection of diluted formalin may protect to some extent a host from subsequent intraperitoneal injection of tumour cells. In the present experiments generalized allergic mechanisms must have been responsible for the rejections since a similar immunity was produced by subcutaneous injection of tumour cells. The fact that BP8 ascitic tumour cells were rejected when given to syngeneic mice by subcutaneous injection is comparable with the result obtained by Apffel and Peters (1967). Subcutaneously injected BP8 ascitic tumour cells seem to induce a rejection phenomenon due to tumour specific antigens and leave antibodies against them in the host when spontaneously rejected.

We have often used formalin-fixed tumour or other tissue cells in the mixed antiglobulin reaction, but the use of formalin-fixed cells for immunization purposes is of interest in the context of possible immunotherapy of cancer.

### SUMMARY

1. C3H mice (originally syngeneic with BP8 tumour cells) may be immunized against BP8 ascitic tumour by repeated intraperitoneal injection of formalin-treated BP8 ascitic cells or by subcutaneous injection of live untreated BP8 cells.

2. In these immunized animals humoral antibodies reactive against BP8 cells could be measured by the mixed antiglobulin reaction. The antibodies appeared at the time the mice showed immunity to tumour challenge.

3. The mixed antiglobulin reaction proved to be much more sensitive than immune cytolysis in detecting these antibodies.

We thank Dr. D. B. Cater for providing us with the BP8 ascites tumour and Mr. B. W. Gurner and Mr. R. H. Matthews for their technical assistance.

The work reported in this paper was undertaken during the tenure of an Eleanor Roosevelt Cancer Fellowship of the American Cancer Society awarded to W. Mori by the International Union Against Cancer.

### REFERENCES

APFFEL, C. A. AND PETERS, J. H.—(1967) *J. natn. Cancer Inst.*, **39**, 1129.
BALDWIN, R. W. AND BARKER, C. R.—(1967) *Br. J. Cancer*, **21**, 793.
CATER, D. B. AND WALDMANN, H.—(1967) *Br. J. Cancer*, **21**, 124.
COOMBS, R. R. A.—(1968) in ' Clinical Aspects of Immunology ' 2nd edition. Edited by Gell, P. G. H. and Coombs, R. R. A. Oxford (Blackwell Scientific Publ.) p. 31.
KLEIN, E. AND KLEIN, G.—(1964) *J. natn. Cancer Inst.*, **32**, 547.
LEJNEVA, O. M., ZILBER, L. A. AND LEVLEVA, E. S.—(1965) *Nature, Lond.*, **206**, 1163.
PASTERNAK, G.—(1965) *J. natn. Cancer Inst.*, **34**, 71.
SELL, K. W. AND SPOONER, R. L.—(1966) *Immunology*, **11**, 533.
SPOONER, R. L., BOWDEN, F. W. AND CARPENTER, R. G.—(1965) *J. Hyg., Camb.*, **63**, 369.
STÜCK, B., BOYSE, E. A., OLD, L. J. AND CARSWELL, E. A.—(1964) *Nature, Lond.*, **203**, 1033.